# Use of preventive measures and serological screening tools for *Leishmania infantum* infection in dogs from Europe

**DOI:** 10.1186/s13071-022-05251-5

**Published:** 2022-05-10

**Authors:** Marta Baxarias, Josep Homedes, Cristina Mateu, Charalampos Attipa, Laia Solano-Gallego

**Affiliations:** 1 Departament de Medicina i Cirurgia Animals, Facultat de Veterinària, Universitat Autònoma de Barcelona, Bellaterra, Spain; 2 Ecuphar Veterinaria SLU, Barcelona, Spain; 3 Department of Tropical Disease Biology, Liverpool School of Tropical Medicine, Liverpool, UK

**Keywords:** Leishmaniosis, Canine, Prevention, Screening diagnostic tools, Europe

## Abstract

**Background:**

There are several screening tools for detecting *Leishmania infantum* infection in dogs and various preventive measures to protect against it. Some studies have investigated them, but not many have described their current use. The aim of this study was to investigate which preventive measures and serological screening tools for *L. infantum* infection were employed from 2012 to 2018 in dogs from different endemic European countries.

**Methods:**

A set of electronic datasheets was completed for each dog from several veterinary centres. Classification of preventive measures included: (1) repellents, (2) vaccines and (3) immunomodulators. Classification of serological tests included the: (1) direct agglutination test (DAT), (2) enzyme-linked immunosorbent assay (ELISA), (3) indirect immunofluorescence (IFI), (4) rapid tests and (5) other assays. Dogs were also classified depending on their risk of exposure and living area.

**Results:**

Information from 3762 dogs was gathered. Preventive measures were applied in 91.5% of dogs and the most frequently used were repellents (86.2%) followed by vaccines (39.8%) and Leisguard^®^ (15.3%). The different types of repellents (collar and spot-on) were used similarly. A combination of a vaccine and repellents was preferred in the high-risk group while the low-risk preferred a combination of Leisguard^®^ and a repellent (Chi-square test: *X*^2^ = 88.41, *df* = 10, *P* < 0.001). Furthermore, all preventive measures were similarly used through the years except for repellents, which were predicted to have a small increase of use each year. Regarding serological screening tools, the most used were rapid and ELISA tests. Rapid tests, ELISA tests and DAT were used similarly through the years, but a significant change was found in the use of IFI and other assays whose use decreased a little each year.

**Conclusions:**

Repellents were the preferred measure, while vaccines and Leisguard^®^ were second-line options. Some dogs were not treated by any measures, which highlights the need for dog owner education. Moreover, there seems to be a preference for rapid tests in the clinical setting to detect specific *L. infantum* antibodies while ELISA or IFI are less often employed. This underlines an increasing problem, as qualitative rapid tests have a variable diagnostic performance limiting the adequate diagnosis of seropositive dogs in endemic areas.

**Graphical abstract:**

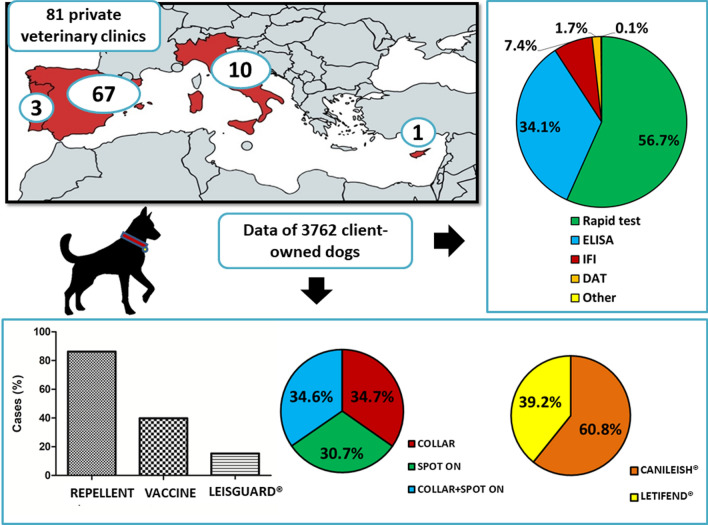

**Supplementary Information:**

The online version contains supplementary material available at 10.1186/s13071-022-05251-5.

## Background

Canine leishmaniosis (CanL) caused by the protozoan *Leishmania infantum* is a zoonotic and endemic disease in the Mediterranean basin [1, 2]. This protozoan is transmitted by the bite of a female phlebotomine sand fly following a digenetic life cycle which consists of two different phases: (i) a promastigote phase, which is an extracellular and motile form that colonizes the middle gut of the sand fly, and (ii) an amastigote phase, which is an intracellular and non-motile form that colonizes macrophages of infected hosts [3, 4]. There are also other potential routes of transmission such as venereal [5, 6], transplacental [6, 7] and through blood transfusion [8, 9], which may play a marginal role compared to the vector transmission [10]. The dog (*Canis lupus familiaris*) is considered the main domestic reservoir for *L. infantum* infection in the Mediterranean basin [2, 10], while other mammals such as wild canids [11], rodents [12] and lagomorphs [13] may be able to maintain a wild life cycle.

The use of preventive measures against *L. infantum* infection has expanded over the last decades [14]. However, there are still two main ways to prevent this infection: (i) physical barriers and insecticides against the vector and (ii) immunoprophylaxis. Regarding the vector, it is recommended to avoid outdoor activities during dawn and dusk (when the vector is highly present), to use fine mesh nets in windows and to use topical insecticides such as synthetic pyrethroid-based compounds, which have both repellent and anti-feeding effects [1, 14, 15]. Topical insecticides are commercially available in different forms: impregnated collars, spot-on and sprays, each of which has different onset and maximum duration [3, 14]. Immunoprophylaxis can be divided into vaccines and immunomodulators. Domperidone (Leisguard^®^) is the only marketed immunomodulator for the prevention of CanL since 2012 [16]. Two commercial vaccines have been available for dogs in Europe: Canileish^®^, which was first launched in 2011 but is not marketed anymore (withdrawn from the market in 2021), and Letifend^®^, which was introduced commercially in 2016 and is currently the only available vaccine in Europe [3, 14, 17].

Moreover, CanL is a complex infection due to its variable clinical manifestations and a wide spectrum of clinical signs and laboratory findings, and several diagnostic techniques are available for its screening and diagnosis [17, 18]. Since a vaccine is available in Europe, serological screening is mandatory prior to vaccination of dogs [17]. In addition, annual screening of dogs is frequently performed in endemic areas to diagnose both dogs progressing towards disease and subclinical infections [10, 17]. The diagnostic methods used in the clinical setting include parasitological diagnosis (direct observation of the parasite), serological techniques (such as ELISA, IFI and rapid chromatographic immunoassay) and molecular techniques (PCR and quantitative PCR) [1, 17, 18].

Some studies have investigated the use of preventive measures in *L. infantum* endemic countries, although their focus was the efficacy and safety of those measures [16, 19, 20] or the veterinary recommendations for their use to dog owners [21–25]. In addition, the development and marketing of new preventive measures such as Letifend^®^ may change the use of the already marketed products. Regarding serological screening tools, several studies have compared their sensitivity and specificity [18, 26, 27] or the use of different types of samples such as saliva [28]. However, the current use of the different preventive measures and serological screening tools available for *L. infantum* infection is relatively unknown. For all these reasons, the aim of this study was to investigate the most used serological screening tools and preventive measures against *L. infantum* infection in dogs from 2012 to 2018 and how their use changed through the years.

## Methods

### Veterinary clinics and cases

**Fig. 1:**
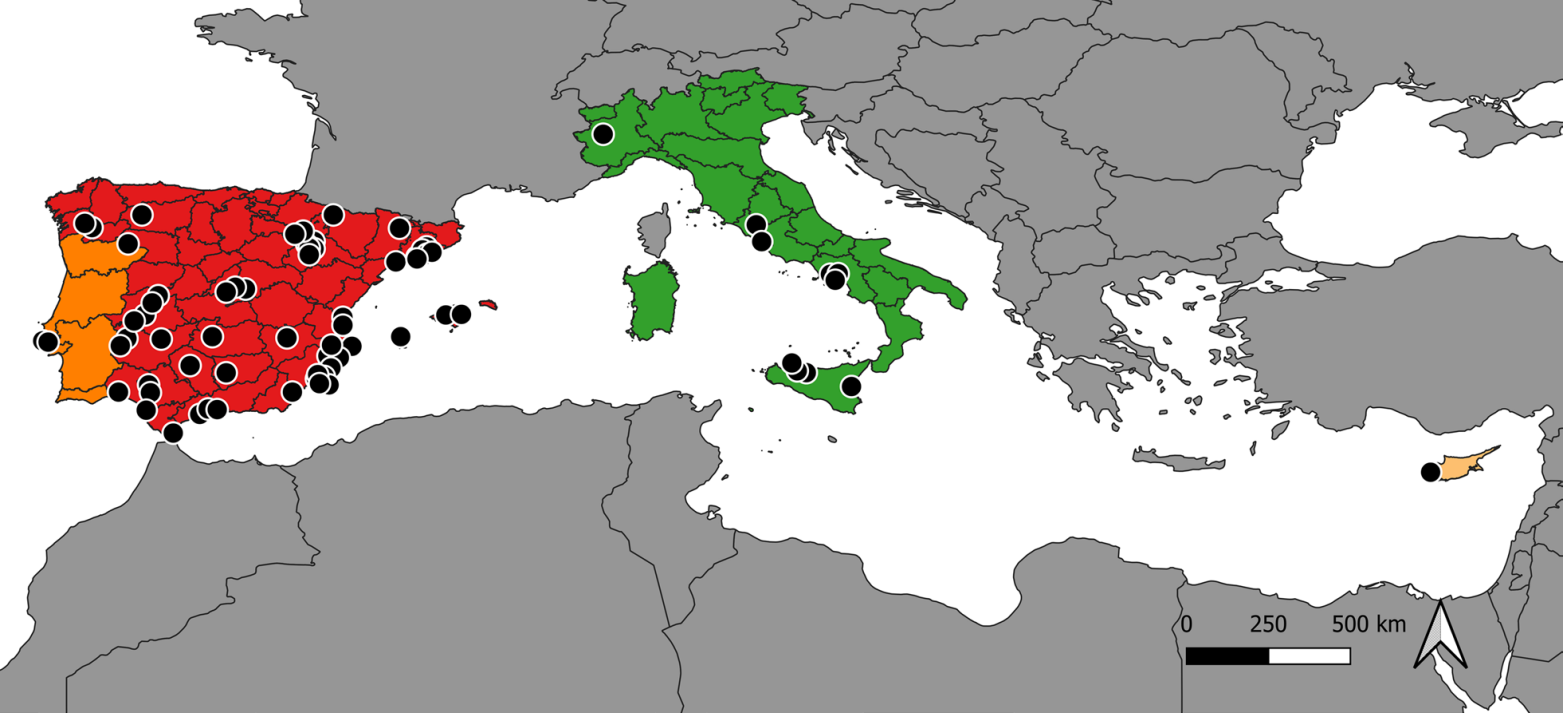
Geographical distribution of all participating veterinary clinics from Europe. Spain is marked in red, Portugal in orange, Italy in green and Cyprus in yellow. Black dots represent each enrolled clinic in each country location

Veterinary clinics from Spain (*n* = 84), Portugal (*n* = 3), Italy (*n* = 17) and Cyprus (*n* = 2), which implemented at least two different preventive measures against *L. infantum* in dogs, were selected for a database search of clinical records by the authors from their contacts and client lists and were contacted to participate. Figure 1 shows the veterinary clinics that enrolled in the study including 67 from Spain, 3 from Portugal, 10 from Italy and 1 from Cyprus. These veterinary clinics provided information of dogs with the following inclusion criteria: (1) apparently healthy dogs and (2) a previous screening serological test for the detection of antibodies against *L. infantum* antigen before the initial use of the preventive measures.

### Study design

Each veterinary clinic received a code to access a website with a set of electronic datasheets that allowed easy data entry. Once the datasheets were completed, their data were automatically uploaded to a common database from which the results were analysed.

The online questionnaire permitted gathering relevant clinical data about dog characteristics (sex, weight, age, breed, risk of exposure and living area) and types of serology tests and preventive measures used. Data of preventive measures were obtained from 2012 to 2017 while data of screening tools were collected from 2012 to 2018.

### Case removal

After collection of cases, removal of inadequate cases was performed. A case was defined as inadequate when: (i) it did not comply with the previously established inclusion criteria or (ii) a duplicate case detected. When a duplicate case was detected, a thorough search was performed to confirm its duplicity as to not lose any information. Information about the same dog with two different preventive measures and non-overlapping timelines was not defined as a duplicate.

### Preventive measures

Dogs were classified considering the combined use of preventive measures. Eleven groups were considered: (i) no preventive measures applied (NON), (ii) only repellents applied (REP), (iii) only Canileish^®^ vaccine (CAN), (iv) only Letifend^®^ vaccine (LET), (v) only Leisguard^®^ (LEI), (vi) Canileish^®^ vaccine + repellent (CAN + REP), (vii) Letifend^®^ vaccine + repellent (LET + REP), (viii) Leisguard^®^ + repellent (LEI + REP), (ix) Canileish^®^ vaccine + Leisguard^®^ (CAN + LEI), (x) Canileish^®^ vaccine + Leisguard^®^ + repellent (CAN + LEI + REP) and (xi) Letifend^®^ vaccine + Leisguard^®^ + repellent (LET + LEI + REP).

Another classification considered the individual use of each product. These four groups were defined as (i) repellent group, which included dogs that used repellent alone or in combination with other products (REP, CAN + REP, LET + REP, LEI + REP, CAN + LEI + REP and LET + LEI + REP), (ii) Canileish^®^, which included dogs that used Canileish^®^ alone or in combination with other products (CAN, CAN + REP, CAN + LEI and CAN + LEI + REP), (iii) Letifend^®^, which included dogs that used Letifend^®^ alone or in combination with other products (LET, LET + REP and LET + LEI + REP), and (iv) Leisguard^®^, which included dogs that used Leisguard^®^ alone or in combination with other products (LEI, LEI + REP, CAN + LEI, CAN + LEI + REP and LET + LEI + REP).

Dogs that used repellent were classified in three different groups based on type of repellent employed: (i) collar, (ii) spot-on and (iii) collar + spot-on.

### Classification of exposure risk and living area

Dogs were classified in two different groups depending on their exposure risk to *L. infantum* infection. High risk was considered when dogs lived outdoors or when dogs that despite living indoors went frequently for a walk in plot of land or forest areas at times when the vector was highly present, for example at dawn and dusk. Low risk classification included those dogs which lived indoors and went only for a walk in urban area or just at times when the vector was barely present.

Another classification depending on living area was also performed. Dogs were classified in three groups: urban area (living in cities or big towns with paved streets and small green areas), periurban area (city outskirts or towns surrounded by large green areas) and rural area (small towns or buildings built far away from human settlements like farms, usually agricultural areas and forests).

### Screening tools

The screening tools were classified in five groups: (i) direct agglutination test (DAT), (ii) enzyme-linked immunosorbent assay (ELISA), (iii) indirect immunofluorescence (IFI), (iv) rapid tests and (5) other assays.

Additionally, a screening campaign by Ecuphar veterinaria SLU was performed in 2018 using Leiscan^®^ and ELISA *in house* [29] to increase the number of enrolled dogs; therefore, a bias was to be expected.

### Statistical analysis

A descriptive study of all collected data was performed. Quantitative variables (age, weight) were assessed using a non-parametric Mann-Whitney *U* test when two groups were compared (high and low risk) while the Kruskal-Wallis *H* test was used when three groups were compared (living area: urban, periurban or rural). Qualitative variables (sex, breed, preventive measures and serological screening tools) were assessed using a Chi-square test. A simple linear regression was calculated to predict the proportion of use for each preventative measure or serological test based on time (from 2012 to 2017 or from 2012 to 2018, respectively).

A *P*-value < 0.05 was considered statistically significant. The Shapiro-Wilk test was performed to detect normal distribution of quantitative variables. The statistical analysis was performed using the package Stats for the software R i386 3.5.1 for Windows. Maps were created using the Free and Open Source QGIS 3.10.4 for Windows. Graphics were plotted using Graphad Prism version 5.00 for Windows.

## Results

### Dog characteristics

Dogs from Spain (3603 dogs), Portugal (64 dogs), Italy (69 dogs) and Cyprus (26 dogs) were enrolled in this study with a total of 3762 dogs. Dog characteristics such as sex, age, weight, breed, risk of exposure and living area are displayed in Table 1. The most common breeds were Yorkshire terrier (7.1%), Labrador retriever (6.7%), German shepherd (6.2%), Maltese (3.9%), Boxer (3.8%), Golden retriever (3.7%) and French bulldog (3.5%).

**Table 1:** Qualitative and quantitative clinical characteristics of the dogs

Qualitative clinical characteristics	N	% (95% CI)
Sex		
Male	2006	53.4 (51.8–55)
Female	1753	46.6 (45–48.2)
Total	3759	
Breed		
Purebred	2711	72.3 (70.9–73.8)
Mixed breed	1037	27.7 (26.2–29.1)
Total	3748	
Risk of exposure		
High	2620	69.9 (68.4–71.4)
Low	1127	30.1 (28.6–31.6)
Total	3747	
Living area		
Urban area	1585	55.5 (53.6–57.3)
Periurban area	818	28.6 (27–30.3)
Rural area	455	15.9 (14.6–17.3)
Total	2858	

Quantitative clinical characteristics	*N*	Mean (± SD)	Minimum	Maximum
Age (years)	3755	7 (± 3.3)	0.5	18.5
Weight (kg)	3753	20 (± 13.7)	1.4	110

*CI* confidence intervals, *N* number of dogs, *SD* standard deviation

No statistically significant differences were found between risk of exposure to the vector (low vs. high risk of exposure) when sex, age and breed were compared. A significant difference (Mann-Whitney test: *U* = 1,876,996, *Z* =  – 13.46, *n*_*1*_ = 2613, *n*_*2*_ = 1125, *P* < 0.0001) was noted when weight was compared between groups of risk of exposure to the vector. Large size dogs (21.9 ± 13.7 kg) were included in the high-risk group while small size dogs (15.7 ± 12.6 kg) were included in the low-risk group.

Quantitative and qualitative characteristics of dogs depending on their living area are listed in Table 2. No differences between groups were found when sex and breed were compared. In the case of age and weight, dogs living in rural areas were younger than dogs living in periurban or urban areas (Kruskal-Wallis *H* test: *X*^2^ = 10.73, *df* = 2, *P* = 0.005) while dogs living in urban areas were smaller in size than dogs living in rural or periurban areas (Kruskal-Wallis *H* test: *X*^2^ = 176.06, *df* = 2, *P* < 0.0001) (Table 2). Moreover, rural area dogs had a higher risk of exposure to *L. infantum* followed by periurban dogs and finally urban dogs (Chi-square test: *X*^2^ = 314.67, *df* = 2, *P* < 0.001).

**Table 2:** Qualitative and quantitative clinical characteristics of the dogs depending on their living area

Qualitative clinical characteristics	Urban area (*N* = 1585)		Periurban area (*N* = 818)		Rural area (*N* = 455)		*P-*value (Chi-square test)
	N	% (95% CI)	N	% (95% CI)	N	% (95% CI)	
Sex							
Male	842	53.1 (50.6–55.6)	461	56.4 (52.9–59.8)	241	53 (48.3–57.6)	0.284
Female	743	46.9 (44.4–49.4)	357	43.6 (40.2–47.1)	214	47 (42.4–51.7)	
Breed							
Purebred	1174	74.1 (71.8–76-2)	576	70.4 (67.2–73.5)	317	69.7 (65.2–73.9)	0.064
Mixed-breed	411	25.9 (23.8–28.2)	242	29.6 (26.5–32.8)	138	30.3 (26.1–34.8)	
Risk of exposure							< 0.001^a^*
High	925	58.4 (55.9–60.8)	676	82.6 (79.9–85.2)	436	95.8 (93.6–97.5)	
Low	660	41.6 (39.2–44-1)	142	17.4 (14.8–20.1)	19	4.2 (2.5–6.4)	

Quantitative clinical characteristics	*N*	Mean (± SD)	N	Mean (± SD)	N	Mean (± SD)	*P-*value (Kruskal-Wallis *H* test)
Age (years)	1585	7.2 (± 3.3)	817	7.1 (± 3.3)	455	6.6 (± 3.1)	0.005^b^*
Weight (kg)	1585	17 (± 13.1)	818	23 (± 13.5)	455	23.9 (± 13.8)	< 0.0001^c^*

*CI* confidence intervals, *N* number of dogs, *SD* standard deviation

^a^*X*^2^ = 314.67, *df* = 2, *P* < 0.001

^b^*X*^2^ = 10.73, *df* = 2, *P* = 0.005

^c^*X*^2^ = 176.06, *df* = 2, *P* < 0.0001

^*^P-value < 0.05 (statistically significant)

### Preventive measures

#### General results

**Fig. 2:**
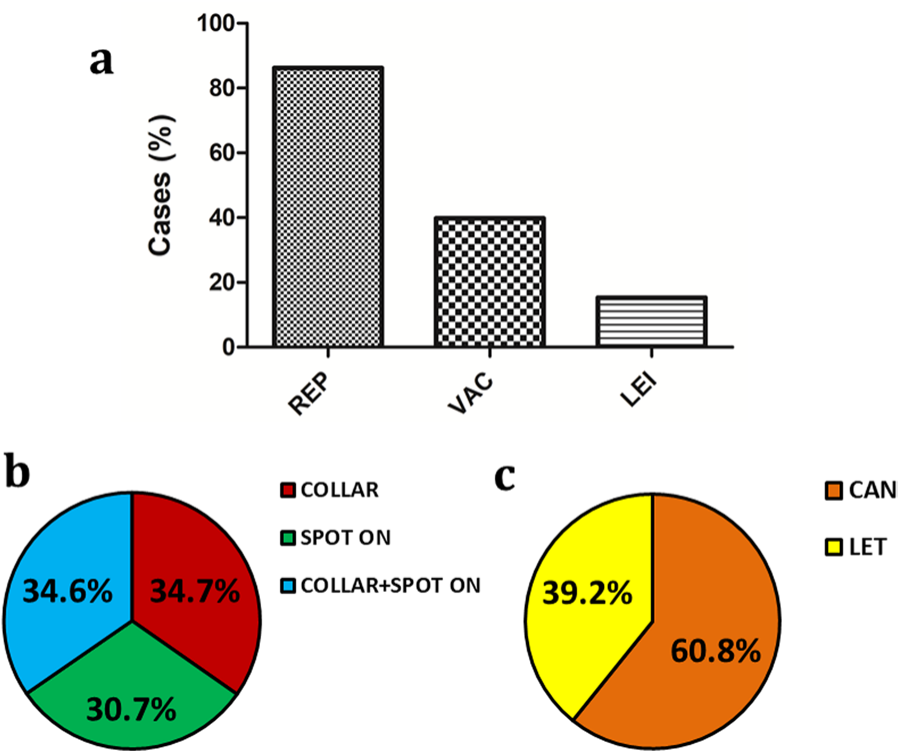
Proportions of (**a**) the individual use of each preventive measure, (**b**) the type of repellent used and (**c**) the vaccine used. Preventive measures represented are repellent group (REP), which included dogs that used repellent alone or in combination with other products, vaccine group (VAC), which included dogs that used vaccine alone or in combination with other products, Leisguard^®^ group (LEI), which included dogs that used Leisguard^®^ alone or in combination with other products, Canileish^®^ group (CAN) and Letifend^®^ group (LET)

Preventive measures were applied for 3444 dogs (91.5%) of all the dogs enrolled. Younger dogs (6.9 ± 3.3 years) were more likely to be treated with preventive measures than older dogs (7.7 ± 3.5 years) (Mann-Whitney test: *U* = 614,890.5, *Z* = –3.79, *n*_*1*_ = 317, *n*_*2*_ = 3438, *P* = 0.0002). The individual use of each preventive measure in the 3444 dogs is plotted in Fig. 2. Repellents (alone or in combination with other products) were the most used preventive measure followed by vaccines (Canileish^®^ or Letifend^®^) and Leisguard^®^ (Fig. 2a). The different types of repellents (collar, spot-on or a combination of both) were used similarly (Fig. 2b) while, in the case of vaccines, Canileish^®^ (60.8%) was more frequently used than Letifend^®^ (39.2%) (Fig. 2c). No statistical differences were observed when the individual use of the different preventive measures depending on sex and breed were compared except for Canileish^®^, which was more often used in purebred dogs (Chi-square test: *X*^2^ = 9.26, *df* = 1, *P* = 0.002) than in mixed-breed dogs. Regarding age, younger dogs were more likely to use repellent (Mann-Whitney test: *U* = 900,141.5, *Z* =  – 2.7, *n*_*1*_ = 518, *n*_*2*_ = 3237, *P* = 0.007), Letifend^®^ (Mann-Whitney test: *U* = 1,084,731, *Z* = -6.42, *n*_*1*_ = 3168, *n*_*2*_ = 587, *P* < 0.0001) or Leisguard^®^ (Mann-Whitney test: *U* = 963,611.5, *Z* = -2.29, *n*_*1*_ = 3184, *n*_*2*_ = 571, *P* = 0.02) than older dogs. As for weight, larger dogs were more likely to use Canileish^®^ (Mann-Whitney test: *U* = 1,213,325, *Z* = –2.72, *n*_*1*_ = 2846, *n*_*2*_ = 907, *P* = 0.006) while smaller dogs were more likely to use Leisguard^®^ (Mann-Whitney test: *U* = 1,043,852.5, *Z* = -5.56, *n*_*1*_ = 3180, *n*_*2*_ = 573, *P* < 0.0001).

**Fig. 3:**
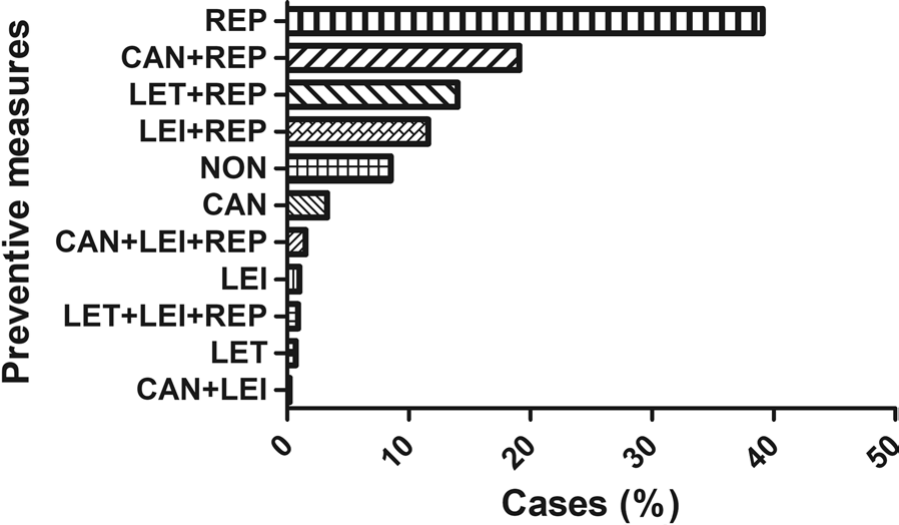
Proportions of preventive measures used against *L. infantum* in all dogs studied. Preventive measures represented are only repellents applied (REP), Canileish^®^ vaccine + repellent (CAN + REP), Letifend^®^ vaccine + repellent (LET + REP), Leisguard^®^ + repellent (LEI + REP), no preventive measures applied (NON), only Canileish^®^ vaccine applied (CAN), Canileish^®^ vaccine + Leisguard^®^ + repellent (CAN + LEI + REP), only Leisguard^®^ applied (LEI), Letifend^®^ vaccine + Leisguard^®^ + repellent (LET + LEI + REP), only Letifend^®^ vaccine applied (LET) and Canileish^®^ vaccine + Leisguard^®^ (CAN + LEI)

Figure 3 shows the combined preventive measures used in all the dogs. The most used preventive measure was repellent alone (Fig. 3). When comparing the proportions of sex, CAN + LEI and CAN + LEI + REP presented the highest proportion of females (58.6%) while REP presented the highest proportion of males (55.9%) (Chi-square test: *X*^2^ = 4.78, *df* = 1, *P* = 0.029), but no other differences were found between the other groups (Table 3). Regarding breed, only CAN + REP was found to have a significantly higher proportion of purebred dogs (77%) when compared to the other preventive measures (44.4%) (Chi-square test: *X*^2^ = 16.53, *df* = 6, *P* = 0.011) (Table 3). When comparing their age, LEI was found to be the oldest group (Table 3). Regarding weight, LEI + REP and LEI were the groups with smaller dogs and significantly different when compared to the other groups (Kruskal-Wallis *H* test: *X*^2^ = 45.82, *df* = 10, *P* < 0.0001) (Table 3).

**Table 3:** Qualitative and quantitative clinical characteristics of the dogs depending on the preventive measures used

Preventive measures	Sex (%, 95% CI)		Breed (%, 95% CI)		Age (years, mean ± SD)	Weight (kg, mean ± SD)	Risk of exposure (%, 95% CI)	
	Male	Female	Purebred	Mixed-breed			High	Low
NON (*N* = 318)	50 (44.4–55.6)	50 (44.4–55.6)	69.1 (63.7–74.1)	30.9 (25.9–36.3)	7.1 (± 3.5)	20 (± 14.3)	71.9 (66.6–76.8)	28.1 (23.2–33.4)
REP (*N* = 1468)	55.9 (53.3–58.5)	44.1 (41.5–46.7)	71.8 (69.4–74.1)	28.2 (25.9–30.6)	7 (± 3.4)	18.8 (± 13.2)	66.4 (63.9–68.8)	33.6 (31.2–36.1)
CAN (*N* = 125)	52 (42.9–61)	48 (39–57.1)	75 (66.4–82.3)	25 (17.7–33.6)	6.5 (± 2.8)	21.8 (± 14.6)	69.4 (60.4–77.3)	30.6 (22.7–39.6)
LET (*N* = 28)	53.6 (33.9–72.5)	46.4 (27.5–66.1)	71.4 (51.3–86.8)	28.6 (13.2–48.7)	4.4 (± 3.7)	19 (± 10.1)	60.7 (40.6–78.5)	39.3 (21.5–59.4)
LEI (*N* = 39)	41 (25.6–57.9)	59 (42.1–74.4)	61.5 (44.6–76.6)	38.5 (23.4–55.4)	8.8 (± 3.3)	11.9 (± 14.1)	43.6 (27.8–60.4)	56.4 (39.6–72.2)
CAN + REP (*N* = 719)	53.8 (50.1–57.5)	46.2 (42.5–49.9)	77 (73.7–80)	23 (20–26.3)	6.3 (± 3.1)	18 (± 14.7)	72.4 (69–75.6)	27.6 (24.4–31)
LET + REP (*N* = 527)	51.4 (47.1–55.8)	48.6 (44.2–52.9)	71.9 (67.9–75.7)	28.1 (24.3–32.1)	5.8 (± 3.3)	19 (± 13.1)	89.9 (87.1–92.4)	10.1 (7.6–13)
LEI + REP (*N* = 436)	52.5 (47.7–57.3)	47.5 (42.7–52.3)	70 (65.5–74.3)	30 (25.7–34.5)	6.1 (± 3.2)	12 (± 13.5)	53.8 (49–58.6)	46.2 (41.4–51)
CAN + LEI (*N* = 9)	22.2 (2.8–60)	77.8 (40–97.2)	44.4 (13.7–78.8)	55.6 (21.2–86.3)	4.4 (± 3.5)	10 (± 13.2)	88.9 (51.8–99.7)	11.1 (0–48.3)
CAN + LEI + REP (*N* = 57)	41.4 (28.6–55.1)	58.6 (44.9–71.4)	75 (61.6–85.6)	25 (14.4–38.4)	6 (± 3)	17.8 (± 15.7)	71.9 (58.5–83)	28.1 (17–41.5)
LET + LEI + REP (*N* = 32)	53.1 (34.7–70.9)	46.9 (29.1–65.3)	68.8 (50–83.9)	31.2 (16.1–50)	6 (± 1.2)	22.5 (± 16.8)	78.1 (60–90.7)	21.9 (9.3–40)
*P*-value	*P* < 0.0001*^a^		*P* < 0.0001*^b^		*P* < 0.0001*^c^	*P* < 0.0001*^d^	*P* < 0.0001*^e^	

*CAN* only Canileish^®^ vaccine, *CAN* + *LE*I Canileish^®^ vaccine + Leisguard^®^, *CAN* + *LEI* + *REP* Canileish^®^ vaccine + Leisguard^®^ + repellent, *CAN* + *REP* Canileish^®^ vaccine + repellent, *CI* Confidence intervals, *LEI* only Leisguard^®^, *LEI* + *REP* Leisguard^®^ + repellent, *LET* only Letifend^®^ vaccine, *LET* + *LEI* + *REP* Letifend^®^ vaccine + Leisguard^®^ + repellent, *LET* + *REP* Letifend^®^ vaccine + repellent, *N* number of dogs, *NON* no preventive measures applied, *REP* only repellents applied, *SD*: standard deviation

^a^Chi-square test: *X*^2^ = 39.63, *df* = 10

^b^Chi-square test: *X*^2^ = 38.72, *df* = 10

^c^Kruskal-Wallis *H* test: *X*^2^ = 84.15, *df* = 10

^d^Kruskal-Wallis *H* test: *X*^2^ = 45.82, *df* = 10

^e^Chi-square test: *X*^2^ = 88.41, *df* = 10

^*^P-value < 0.05 (statistically significant)

Additional file 1: Fig. S1 shows the use of the different marketed brands of each type of repellent: collar (Additional file 1: Fig. S1a) and spot-on (Additional file 1: Fig. S1b). The most used products were the Scalibor^®^ collar (70%) and the Advantix^®^ spot-on (61%). Significant differences were found regarding breed, age and weight. In detail, purebred dogs used more frequently a combination of both collar and spot-on, while mixed-breed dogs used collars alone more frequently (Chi-square test: *X*^2^ = 8.03, *df* = 2, *P* = 0.018). Dogs using collars alone were younger (6.8 years) than dogs using spot-on alone (7.1 years) (Kruskal-Wallis H test: *X*^2^ = 6.27, *df* = 2, *P* = 0.044) while dogs using spot-on alone were smaller in size (14.5 kg) than dogs using collars alone (22.2 kg) or a combination of collar and spot-on (22.5 kg) (Kruskal-Wallis H test: *X*^2^ = 299.11, *df* = 2, *P* < 0.0001).

#### Preventive measures by risk of exposure

The use of preventive measures against *L. infantum* was similar when risk of exposure was compared (91.3% high-risk group and 92.1% low-risk group). Letifend^®^ was used more frequently in the high-risk group (Chi-square test: *X*^2^ = 107.02, *df* = 1, *P* < 0.001) while Leisguard^®^ was used more often in the low-risk group (Chi-square test: *X*^2^ = 54.69, *df* = 1, *P* < 0.001). Regarding the type of repellents used, the high-risk group had a higher rate of using both types of repellents together (collar and spot-on) while the low-risk group had a higher rate of using collar or spot-on alone (Chi-square test: *X*^2^ = 92.80, *df* = 2, *P* < 0.001).

Most of the preventive measures were more frequently used in the high-risk group except for LEI + REP and LEI, which were similarly used in both groups. In fact, LEI + REP and LEI were found to have a significantly higher proportion of use in the low-risk of exposure group than other preventive measures (Chi-square test: *X*^2^ = 88.41, *df* = 10, *P* < 0.0001) (Table 3). On the other hand, LET + REP was found to have the highest proportion of use in the high-risk group and was significantly different when compared to the other groups (Table 3).

#### Preventive measures by living area

**Fig. 4:**
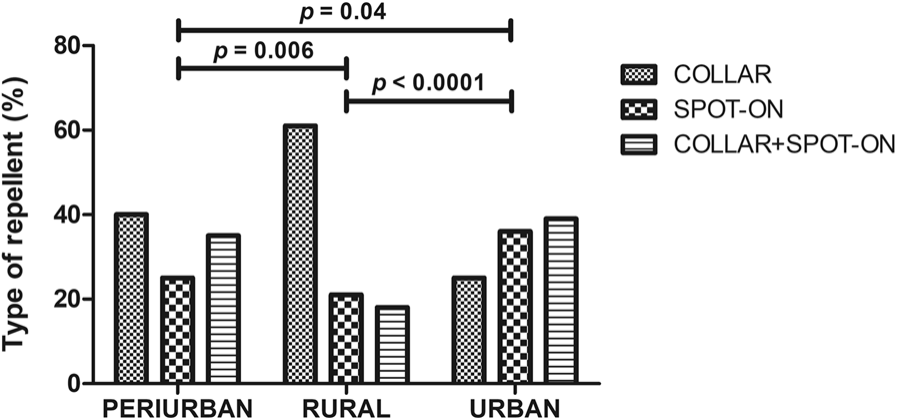
Proportions of the type of repellent used depending on the living area. Statistical significance was found in the following comparisons: Periurban vs. rural (Chi-square test: *X*^2^ = 10.01, *df* = 2, *P* = 0.006) and urban areas (Chi-square test: *X*^2^ = 6.07, *df* = 2, *P* = 0.04) and rural vs. urban areas (Chi-square test: *X*^2^ = 26.75, *df* = 2, *P* < 0.0001)

Preventive measures were applied differently depending on the living area showing a higher rate of use in urban area (93.2%) followed by periurban (91.6%) and rural (87.9%) areas (Chi-square test: *X*^2^ = 13.34, *df* = 2, *P* = 0.001). The use of collar, spot-on and a combination of both was also compared between urban, periruban and rural areas and significant differences were found (Chi-square test: *X*^2^ = 194.23, *df* = 4, *P* < 0.001) with a higher use of collar alone in rural and periruban areas while a combination of both collar and spot-on was preferred in urban areas (Fig. 4).

Furthermore, REP was the preventive measure used at the most similar frequency in all areas with 47.5% of use in the urban followed by 30.4% in the periurban and 22.1% in the rural areas. CAN + REP and LET + REP were significantly more used in urban areas with 64% and a 78% frequency, respectively (Chi-square test: *X*^2^ = 170, *df* = 20, *P* < 0.0001). Moreover, LET + REP was significantly more used in urban areas than CAN + REP (Chi-square test: *X*^2^ = 30.35, *df* = 2, *P* < 0.001).

#### Preventive measures trends

**Fig. 5:**
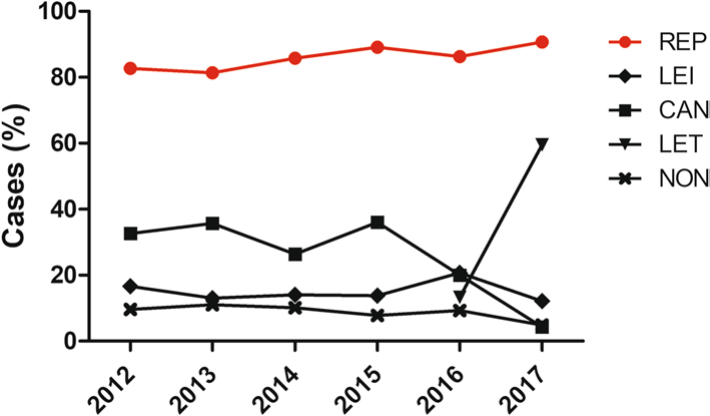
Proportions of the use of the different products through the years studied (2012–2017). Preventive measures represented are repellent group (REP), which included dogs that used repellent alone or in combination with other products, Leisguard^®^ group (LEI), which included dogs that used Leisguard^®^ alone or in combination with other products, Canileish^®^ group (CAN), which included dogs that used Canileish^®^ alone or in combination with other products, Letifend^®^ group (LET), which included dogs that used Letifend^®^ alone or in combination with other products, and no preventive measures applied (NON). Data in red present a significant regression: REP (F(1,4) = 12.15, *P* = 0.0252)

The use of the different products from 2012 to 2017 is plotted in Fig. 5. Repellents were the most used always by > 80% of the dogs studied (Fig. 5). A significant regression was only found in the use of repellents with an R^2^ of 0.75 (Fig. 5). The predicted use of repellents was equal to -3252.31 + 1.66 of percentage of the use of repellents when time was measured in years, so the percentage of use of repellents increased 1.66% for each year.

### Serological screening tools

#### General results

**Fig. 6:**
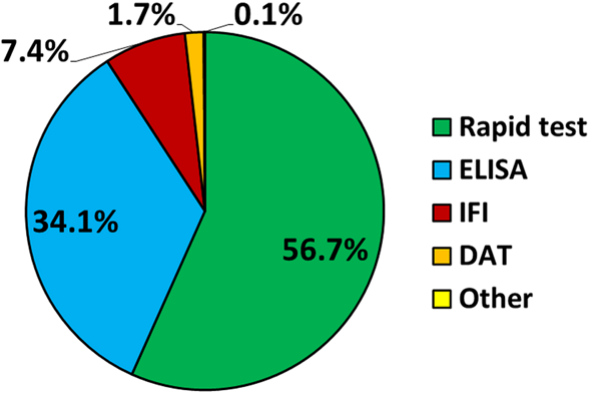
Proportions of the different types of serological screening tests. Screening tools represented are the direct agglutination test (DAT), enzyme-linked immunosorbent assay (ELISA), indirect immunofluorescence (IFI), rapid tests and other assays

The different types of serological screening tests employed are shown in Fig. 6 while the different brands of serological screening tests are shown in Additional file 2: Fig. S2. Rapid tests were the most used (SNAP-Idexx) followed by ELISA tests (Leiscan^®^). IFI and DAT were used in < 10% of the cases (Fig. 6, Additional file 2: Fig. S2).

#### Screening tools trends

**Fig. 7:**
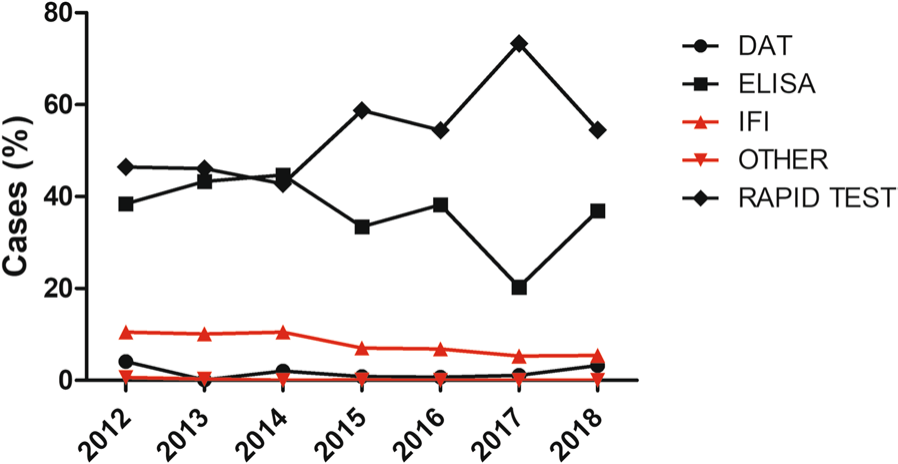
Proportions of the use of the different types of serological screening tests through the years studied (2012–2018). Screening tools represented are direct agglutination test (DAT), enzyme-linked immunosorbent assay (ELISA), indirect immunofluorescence (IFI), rapid tests and other assays. Data in red present a significant regression: IFI (F(1,5) = 35.08, *P* = 0.002) and other (F(1,5) = 9.23, *P* = 0.0288)

The use of the different types of serological screening tests from 2012 to 2018 is displayed in Fig. 7. Rapid tests followed by ELISA were the most frequently used techniques (Fig. 7). A significant regression was found on the use of IFI tests and other tests with an R^2^ of 0.88 and 0.65, respectively. The predicted use of IFI tests was equal to 2066.12—1.02 of percentage of the use of IFI tests when time is measured in years, so the percentage of use of IFI tests decreased 1.02% for each year. The predicted use of other tests was equal to 172.86–0.09 of percentage of the use of other tests when time was measured in years, so the percentage of use of other tests decreased 0.09% for each year.

## Discussion

Previous studies have investigated the veterinary recommendations for the use of preventive measures to dog owners in Spain and other European countries and found out that most veterinarians recommend preventive measures against *L. infantum* to their clients [21–25]. These recommendations can be linked directly to the results of the present study as at least one preventive measure was applied in > 90% of the dogs. Furthermore, veterinary recommendations seem to prioritize the use of repellents over vaccines or Leisguard^®^ [22, 23], which is also highlighted by the results of the present study where a repellent was used in > 80% of the dogs while vaccines and Leisguard^®^ were used by < 50% throughout all years studied. As expected, these recommendations are in line with the published guidelines [14], which endorse the use of repellents in both endemic and fringe areas, while vaccines and Leisguard^®^ are described as optional.

Regarding repellent brands, a previous study [23] reported that the most frequently recommended were Seresto^®^, Advantix^®^ and Scalibor^®^. Both the present study and an additional study [19] showed similar results with the most used collar being Scalibor^®^ while Advantix^®^ was the most used spot-on. Interestingly, a study performed in north-eastern Spain [22] described a preference for recommending collars (98% of the veterinarians recommended collars to their clients) over spot-on (67% of the veterinarians recommended spot-on), in disagreement with the present results in which there was no difference between the use of collar or spot-on, although the reason for these results could be related to the higher use of collars in periurban and rural areas compared to urban areas found in this study. Regarding vaccines, Montoya et al. [23] reported a higher use of Letifend^®^ than Canileish^®^. However, the present study differs as a higher use of Canileish^®^ was found when compared with Letifend^®^. This discrepancy is due to the fact that data on dogs were included from 2012 when Canileish^®^ was still on the market and Letifend^®^ was not marketed yet [3, 14, 17].

Interestingly, Leisguard^®^ was more frequently administered to smaller dogs [19], as observed in this study. One of the reasons for this result is that the Leisguard^®^ dose administration is linked to body weight so large dogs need a high daily dose and therefore a higher expenditure than when being used for small dogs [16]. Another explanation is the fact that small size dogs are more prone to adverse effects after vaccination [30, 31].

An association between socioeconomic status of the dog owner and CanL has been previously documented [32]. Owners with a low income cannot afford some products and that may affect the disease control and even the nutrition and survival of the dog [32]. The presence of a backyard at the residence with a predominance of land and/or vegetation was also associated with CanL [32], which could be a consequence of not only an environmental factor but also of the smaller use of preventive measures in periurban and rural areas as described in the present study, among other factors. Another study from Brazil [33] went further and associated CanL with not just rural areas (small farms) but also the larger size of the dogs (usually used as guard dogs) and lack of owner knowledge about CanL. Coincidentally, in this study, larger dogs were more frequently classified in the high-risk exposure group and living in rural or periurban areas, which could explain its association with CanL.

The use of screening tools was also widespread as stated previously by other studies [19, 22–25]. Concerning serological tests, rapid tests and ELISA seem to be preferred by clinicians in the present study as previously reported [19, 22–25]. Rapid tests (56.7%) are being used more in the clinical setting probably because of their fast results, low price and easy performance, while other types of tests such ELISA (34.1%) and IFI (7.4%) are employed less because of increased time of performance and mainly because they need to be conducted in laboratories by trained personnel. However, ELISA is used more than IFI because IFI’s interpretation is subjective and its result depends on the operator’s experience and skill to interpret the test while ELISA is interpreted objectively using an ELISA reader to quantify the result [26]. These results highlight an increasing problem in the clinical setting as qualitative rapid tests have a good specificity but are less sensitive than quantitative laboratory tests such as IFI and ELISA and therefore rapid tests can misdiagnose seropositive cases [10, 17, 18, 34]. It is important to remark that rapid tests have a low sensitivity in detecting apparently healthy seropositive dogs [26]. This fact is extremely concerning when testing apparently healthy infected dogs as further investigations will not be performed and therefore infection will not be detected.

The limitations of the study are that, even as the study was expected to collect information from different countries, a limited number of dogs from Portugal, Italy and Cyprus were included, so the information received was mainly from Spain. Furthermore, just a small sample of the vast dog population of Spain (> 7.5 million registered dogs) [35] was included and the use of preventive measures might be overestimated.

## Conclusions

In conclusion, dog owners in Spain follow the veterinarian’s recommendations for the use of preventive measures against *L. infantum* infection as endorsed by the published guidelines. Repellents were the preferred measure, while vaccines and Leisguard^®^ were second-line options. However, there are still dogs that do not use preventive measures in endemic regions. Regarding serological screening tools, there seems to be a preference for the use of rapid tests in the clinical setting to detect specific *L. infantum* antibodies while other types of tests such ELISA and IFI are less often employed. The results of this study reinforce the need to sensitize owners about the importance of protecting dogs against the parasite and clinicians about the limitations that qualitative serological techniques can present in the diagnosis of seropositive animals in endemic areas.

## Supplementary Information

**Additional file 1: Figure S1.** Proportions of **a**) the use of collar marketed brands and **b**) the use of spot-on marketed brands in all dogs studied.

**Additional file 2: Figure S2.** Proportions of the different brands of serological screening tests.

## Data Availability

The datasets analysed during the current study are available from the corresponding author on reasonable request.

## Abbreviations

CAN: Only Canileish^®^ vaccine
CanL: Canine leishmaniosis
CAN + LEI: Canileish^®^ vaccine + Leisguard^®^
CAN + LEI + REP: Canileish^®^ vaccine + Leisguard^®^ + repellent
CAN + REP: Canileish^®^ vaccine + repellent
DAT: Direct agglutination test
ELISA: Enzyme-linked immunosorbent assay
IFI: Indirect immunofluorescence
LEI: Only Leisguard^®^
LEI + REP: Leisguard^®^ + repellent
LET: Only Letifend^®^ vaccine
LET + LEI + REP: Letifend^®^ vaccine + Leisguard^®^ + repellent
LET + REP: Letifend^®^ vaccine + repellent
NON: No preventive measures applied
PCR: Polymerase chain reaction
REP: Only repellents applied
