# Supplementary figures and images for: Use of preventive measures and serological screening tools for Leishmania infantum infection in dogs from Europe
Source: Parasit Vectors. 2022 May 10;15:134. doi: 10.1186/s13071-022-05251-5 (PMC9088038; doi:10.1186/s13071-022-05251-5)

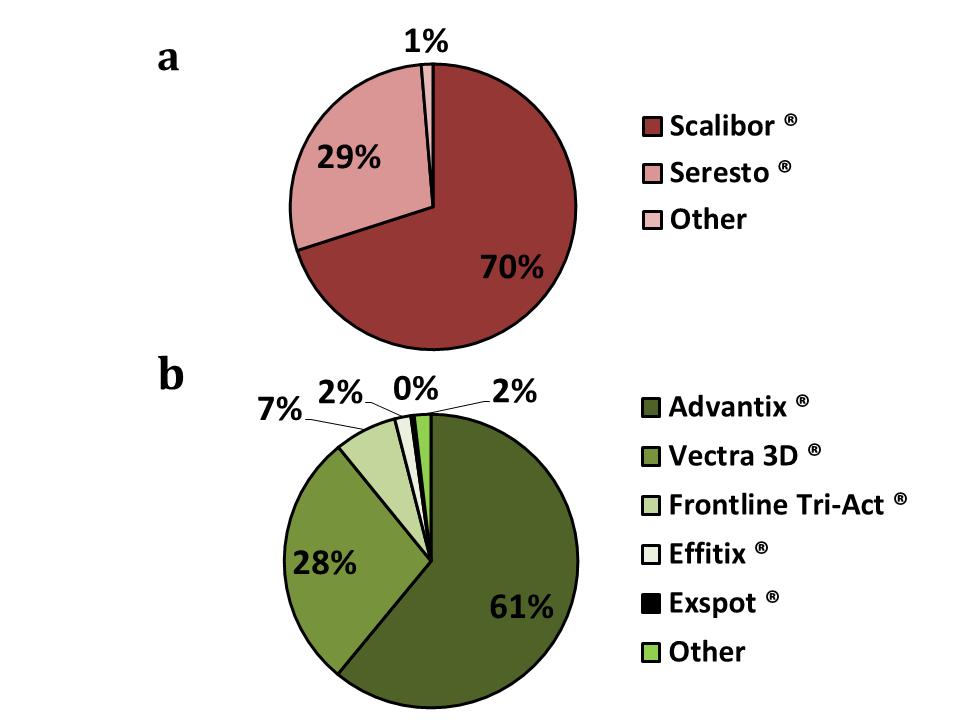

Supplement: Supplementary file 1 — Additional file 1: Figure S1. Proportions of a) the use of collar marketed brands and b) the use of spot-on marketed brands in all dogs studied. [file 13071_2022_5251_MOESM1_ESM.tif]

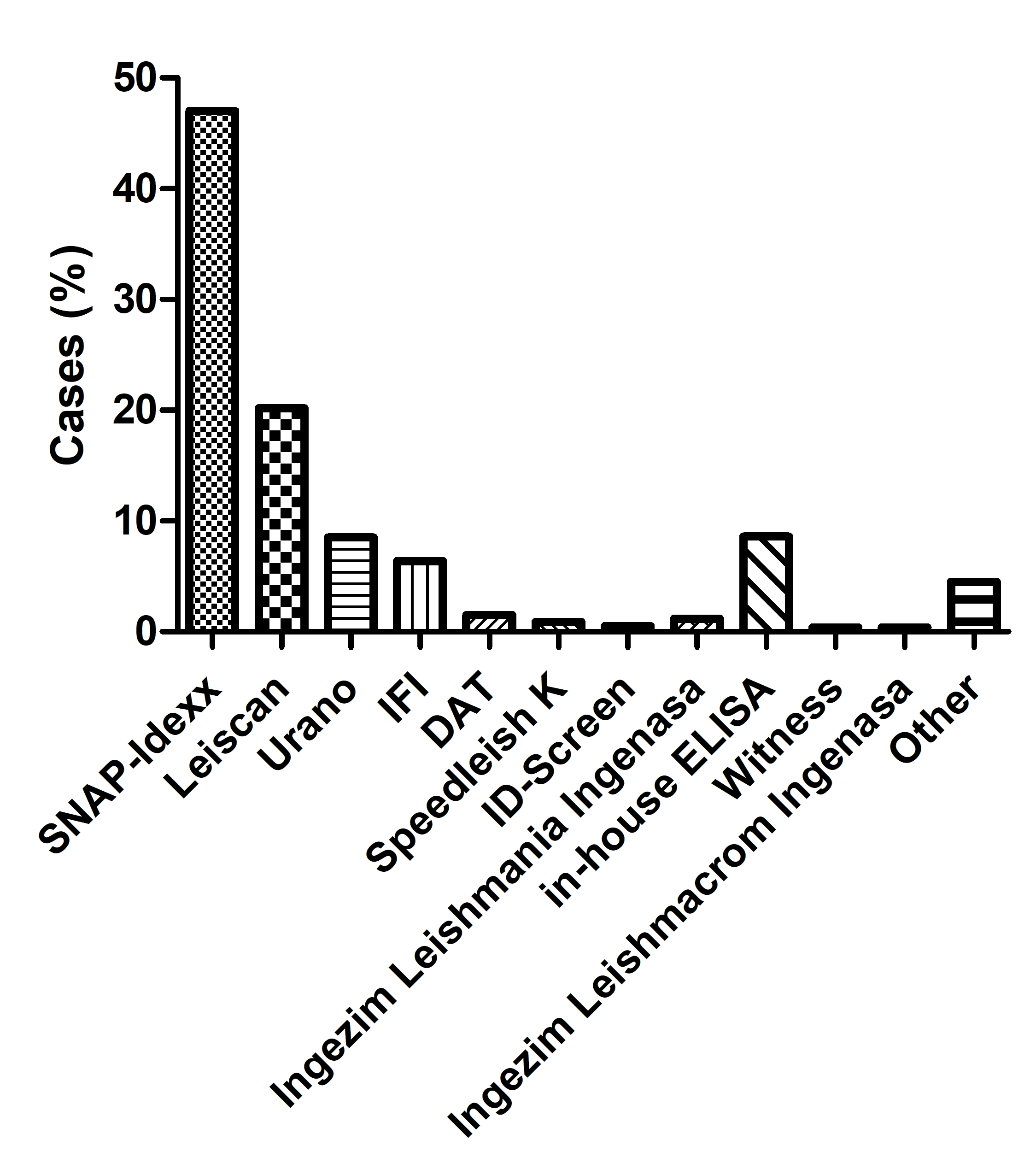

Supplement: Supplementary file 2 — Additional file 2: Figure S2. Proportions of the different brands of serological screening tests. [file 13071_2022_5251_MOESM2_ESM.tif]
